# Unimolecular Reactions of 2,4-Dimethyloxetanyl Radicals

**DOI:** 10.1021/acs.jpca.2c08290

**Published:** 2023-03-10

**Authors:** Anna C. Doner, Judit Zádor, Brandon Rotavera

**Affiliations:** ^†^Department of Chemistry and ^§^College of Engineering, University of Georgia, Athens, Georgia 30602, United States; ‡ Combustion Research Facility, Sandia National Laboratories, Livermore, California 94550, United States

## Abstract

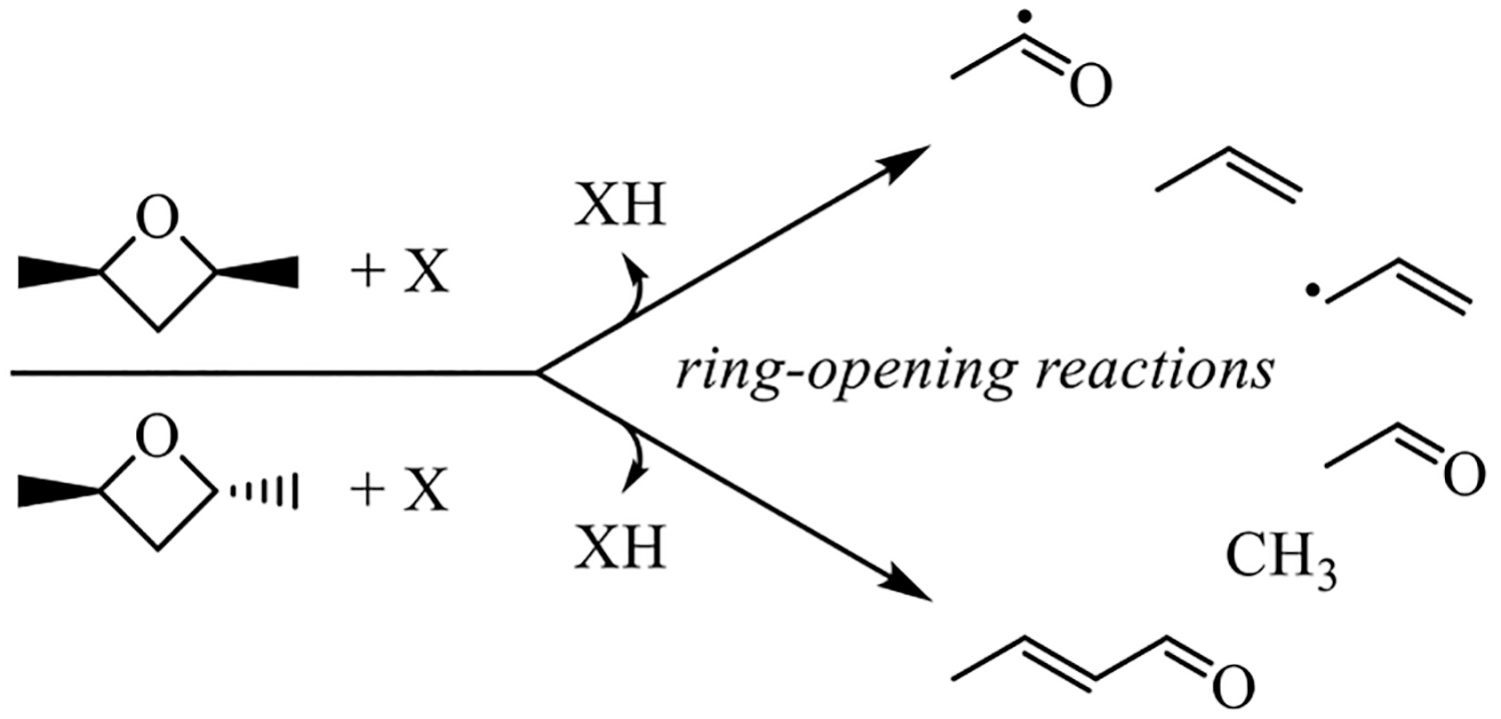

Alkyl-substituted oxetanes are cyclic ethers formed via unimolecular reactions of QOOH radicals produced via a six-membered transition state in the preceding isomerization step of organic peroxy radicals, ROO. Owing to radical isomer-specific formation pathways, cyclic ethers are unambiguous proxies for inferring QOOH reaction rates. Therefore, accounting for subsequent oxidation of cyclic ethers is important in order to accurately determine rates for QOOH → products. Cyclic ethers can react via unimolecular reaction (ring-opening) or via bimolecular reaction with O_2_ to form cyclic ether-peroxy adducts. The computations herein provide reaction mechanisms and theoretical rate coefficients for the former type in order to determine competing pathways for the cyclic ether radicals. Rate coefficients of unimolecular reactions of 2,4-dimethyloxetanyl radicals were computed using master equation modeling from 0.01 to 100 atm and from 300 to 1000 K. Coupled-cluster methods were utilized for stationary-point energy calculations, and uncertainties in the computed rate coefficients were accounted for using variation in barrier heights and in well depths. The potential energy surfaces reveal accessible channels to several species via crossover reactions, such as 2-methyltetrahydrofuran-5-yl and pentanonyl isomers. For the range of temperature over which 2,4-dimethyloxetane forms during *n*-pentane oxidation, the following are the major channels: 2,4-dimethyloxetan-1-yl → acetaldehyde + allyl, 2,4-dimethyloxetan-2-yl → propene + acetyl, and 2,4-dimethyloxetan-3-yl → 3-butenal + methyl, or, 1-penten-3-yl-4-ol. Well-skipping reactions were significant in a number of channels and also exhibited a markedly different pressure dependence. The calculations show that rate coefficients for ring-opening are approximately an order of magnitude lower for the tertiary 2,4-dimethyloxetanyl radicals than for the primary and secondary 2,4-dimethyloxetanyl radicals. Unlike for reactions of the corresponding ROO radicals, however, unimolecular rate coefficients are independent of the stereochemistry. Moreover, rate coefficients of cyclic ether radical ring-opening are of the same order of magnitude as O_2_ addition, underscoring the point that a competing network of reactions is necessary to include for accurate chemical kinetics modeling of species profiles for cyclic ethers.

## Introduction

During autoxidation below ∼900 K, organic molecules proceed through a series of bimolecular and unimolecular reaction steps that are sensitive to molecular structure and are critical to the ignition properties of hydrocarbons.^1^ One of the key species formed in the incipient stages of autoxidation is QOOH^2−4^ - carbon-centered radical isomers of substituted peroxy radicals, ROO, which are the primary products of organic radical reactions with O_2_. QOOH radicals either react with another O_2_ molecule or decompose unimolecularly and, crucially, control chain-propagating and chain-branching processes. While several competing channels unfold during the course of reaction, the most important products from the bimolecular O_2_ addition step are ketohydroperoxides,^5^ while the most characteristic unimolecular products of QOOH are cyclic ethers.

Owing to extreme difficulty in experimental detection,^2−4^ especially in reacting systems, immediate decomposition products are relied upon to infer the presence and concentration of QOOH radicals.^6,7^ For instance, concentration profiles of cyclic ethers are linked to that of the parent QOOH radicals. While the detection of cyclic ethers using, for instance, VUV photoionization mass spectrometry^8,9^ and VUV absorption^10,33^ is well established, the correct inference of QOOH concentrations requires that the loss mechanisms of these cyclic ethers are also well characterized and accounted for in chemical kinetics modeling.

**Figure 1:**
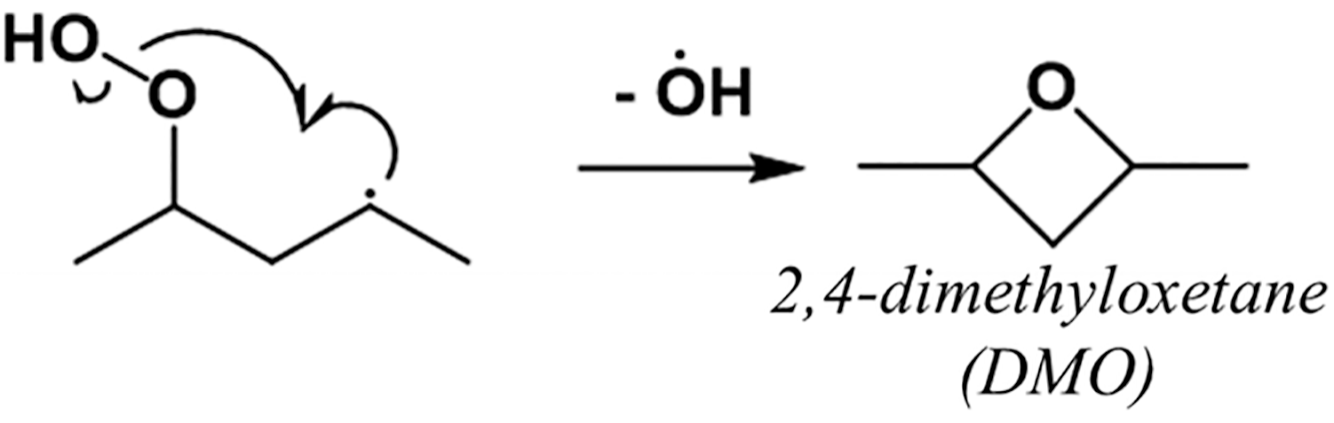
2,4-Dimethyloxetane is formed through cyclic ether formation from the QOOH, 2-hydroperoxypentan-4-yl, which is formed in the low-temperature combustion of *n*-pentane.

As an example, 2,4-dimethyloxetane (DMO) is the second-most-abundant alkyl-substituted cyclic ether produced in *n*-pentane combustion experiments according to Bugler et al.^11^ (Figure 1). The combustion mechanism from Bugler et al.^12^ provides theoretical rate coefficients for the formation of DMO from the corresponding QOOH radical, 2-hydroperoxy-pentan-4-yl, which significantly improved agreement between experimental and modeled species profiles of DMO when compared to rate coefficients calculated based on barrier heights computed at the CBS-QB3 level of theory.^13^ The resulting agreement of the model with the experiments improved. However, only four consumption reactions for DMO were prescribed using estimated rate coefficients. As is common in chemical kinetics mechanism development for all types of hydrocarbons and biofuels, Bugler et al.^12^ combined H-abstraction, ring-opening, and β-scission into one step, varying only by the H-abstractor (OH or HO_2_) and which two bonds are broken (Table 1). Therefore, the balance of production and consumption of DMO, which determines steady state concentration, requires detailed characterization in order to reduce the outstanding uncertainties and further constrain the models.

**Table 1:**
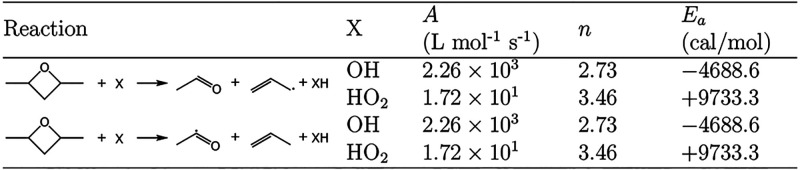
Consumption Reactions Prescribed by Bugler et al.^11^ for 2,4-Dimethyloxetane Combine H-Abstraction and Scission of Two Bonds into One Step with an Estimated Rate Coefficient^a^

a The rate coefficients differ only by the H-abstracting radical.

Examining the elementary reactions of DMO in low-temperature combustion can reveal additional products or bimolecular reactions. Doner et al.^14^ studied reactions of DMO radicals with O_2_ and revealed a plethora of reactions that aid in refining the interpretation of experimental data. For example, the peroxy radicals derived from DMO alter the balance of OH and HO_2_ and either enhance or inhibit ignition, respectively. The products of unimolecular decomposition of alkyl-substituted cyclic ether peroxy radicals also significantly depended on the stereochemistry of the radicals.^14,15^

**Figure 2:**
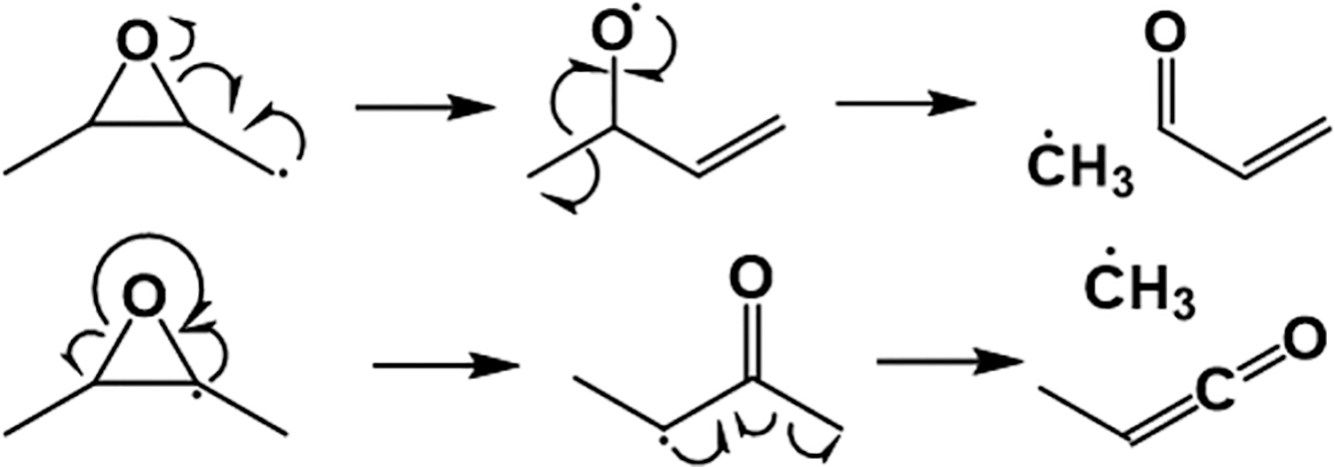
Proposed pathways for the decomposition of the primary and tertiary 2,3-dimethyloxirane radicals from Doner et al.^15^ both involve ring-opening and then β-scission.

Experimental kinetics data for oxidation and pyrolysis is available for the analogous alkyl-substituted three- and five-membered cyclic ethers, *cis*- and *trans*-2,3-dimethyloxirane^15^ and 2,5-dimethyltetrahydrofuran,^16,17^ respectively. Ring-opening of the primary 2,3-dimethyloxiranyl radical leads to an unsaturated alkoxy radical, which decomposes to acrolein and methyl, as shown in Figure 2. Ring-opening of the tertiary 2,3-dimethyloxiranyl radical leads to a resonance-stabilized carbonyl radical, 1,2-dimethylvinoxy, which decomposes to give methylketene and methyl (Figure 2).

**Figure 3:**
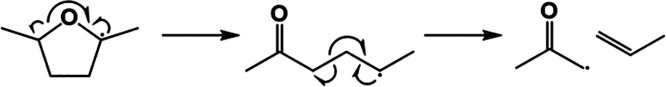
Pathway for unimolecular decomposition of 2,5-dimethyltetrahydrofuran-2-yl proposed by Simmie is ring-opening followed by β-scission.^16^

Due to the larger cyclic ether ring size of DMO, such resonance stabilization is not present for ring-opened tertiary DMO radicals (*vide infra*). Similarly, the tertiary 2,5-dimethyltetrahydrofuranyl radical ring-opens to produce a non-resonance-stabilized carbonyl radical, 2-hexanone-5-yl, which undergoes β-scission, producing acetonyl and propene as shown in Figure 3.

The pathway in Figure 3 is supported by modeling and speciation measurements from Fenard et al.^17^ Although ring-opening via C–C bond scission incurs consistently higher energy barriers compared to ring-opening via C–O bond scission, these pathways are sometimes relevant at low temperatures. For example, vinyl acetate was detected at 650 K in experiments on the oxidation of 2,3-dimethyloxirane stereoisomers in Doner et al.^15^

Simmie^16^ noted that 2,5-dimethyltetrahydrofuran has two conformers, *cis* and *trans*, but did not treat them separately because they are nearly isoenergetic. However, Doner et al.^15^ showed that the diastereomers of 2,3-dimethyloxiranyl radicals and 2,3-dimethyloxiranyl peroxy radicals are also nearly isoenergetic yet produce significantly different experimental branching fractions.

The present work employs KinBot^18,19^ to automate the mapping of potential energy surfaces for unimolecular reactions of 2,4-dimethyloxetanyl radicals, including stereoisomers. The potential energy surfaces are explored using barrier height and branching fraction cutoff criteria, which allowed us to include bimolecular product channels from species formed via ring-opening products of the cyclic ether radicals, e.g., *anti*-2,4-dimethyloxetan-1-yl → pent-1-en-4-oxy → allyl + acetaldehyde, in order to infer reaction pathways and compare with prescribed product channels in chemical kinetics mechanism of *n*-pentane. Coupled-cluster methods are then utilized to calculate electronic energies for all stationary points, which are then incorporated into master equation calculations to determine rate coefficients from 300 to 1000 K and from 0.01 to 100 atm. Branching fractions are then extracted at 1 atm over a reaction time of 20 ms. The main objective herein is to expand on similar computations for 2,4-dimethyloxetanyl-peroxy radical isomers^14^ by producing theoretical rate coefficients and insight into unimolecular reactions of 2,4-dimethyloxetanyl. The broader aim is to expand the level of detail included in chemical kinetics mechanisms, which is important because cyclic ethers are the closest proxies for modeling unimolecular reaction rates of QOOH.

## Computational Methods

The subsequent sections outline the methods utilized to produce potential energy surfaces for unimolecular reaction of the cyclic ether radicals in Figure 4 and the method for computing rate coefficients with quantified uncertainties (Figures S1–S10).

**Figure 4:**
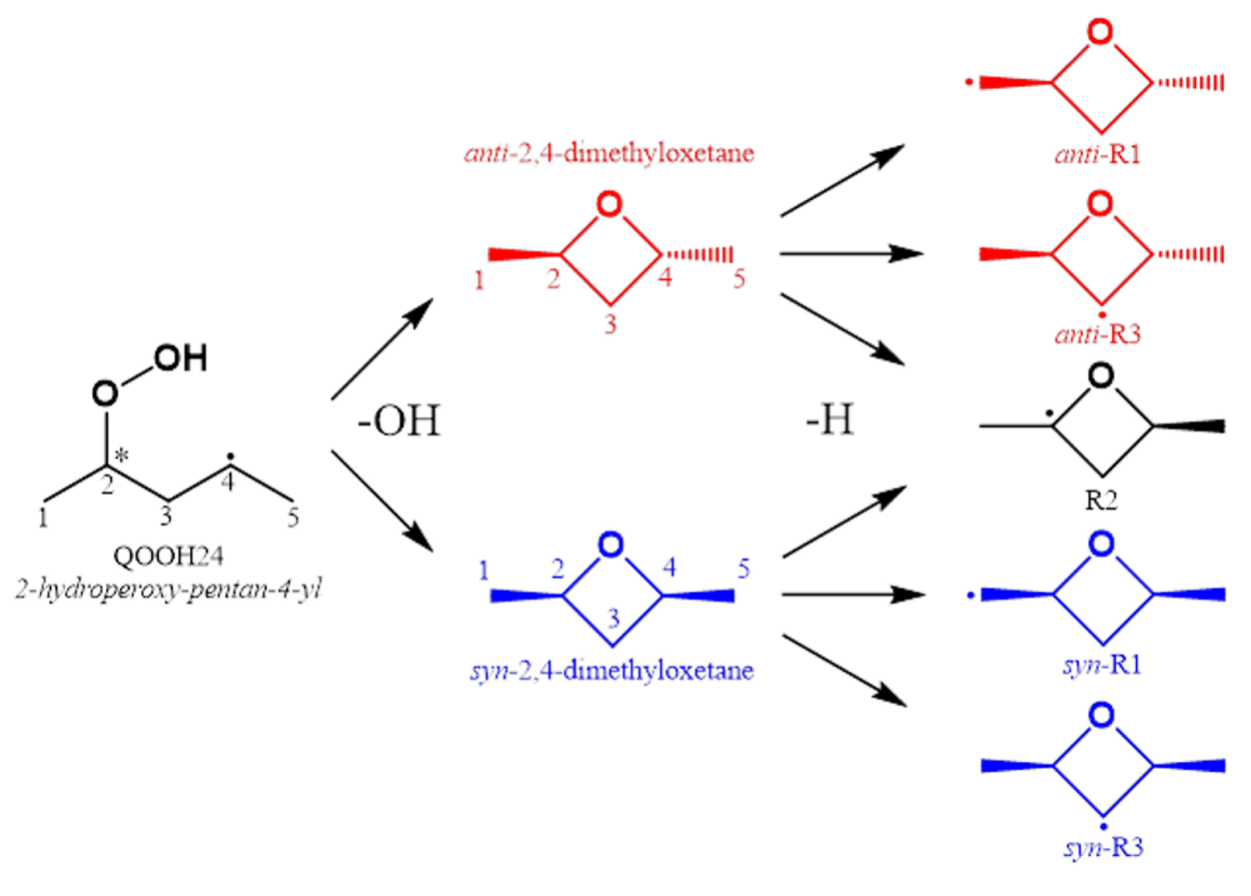
2,4-Dimethyloxetane (DMO) is formed from the QOOH species, 2-hydroperoxy-pentan-4-yl, in the low-temperature combustion of *n*-pentane. DMO has two diastereomers, *syn*- and *anti*-DMO, where *syn* and *anti* refer to the orientation of the methyl groups relative to the ether group. H-abstraction from both diastereomers can yield five unique radicals. The radicals are named as the methyl group orientation followed by R, followed by the carbon number where the radical is centered.

### Potential Energy Surfaces

Potential energy surfaces for each of the five DMO radicals shown in Figure 4 were explored automatically with the open-source kinetics workflow code, KinBot.^18−20^ The initial saddle point guess is constructed by a series of constrained optimization steps at the L0 = AM1 level of theory. The initial guess is refined to a true first-order saddle-point (FOSP) at the L1 = B3LYP/6-31+G level of theory and confirmed by intrinsic reaction coordinate (IRC) calculations at the same level. The conformational searches were also performed at the L1 level. The conformational search was performed on a 60° (six-point) grid. Ring conformers were generated by systematically distorting the backbone of the ring. When determining the number of ring conformers to generate, the following rules were used. For three-membered rings, no ring conformers were generated. For four-membered, five-, and six-membered rings, the number of trial ring conformers was calculated as , where *n*_ring_ is the size of the ring. For fused rings, the size of the smallest complete ring is taken. The conformers of each subring are sampled as usual. Finally, the conformers of the acyclic side chains of each ring conformer are then sampled on the 60° grid, yielding  total trial conformers, where *N* is the number of subrings. However, if the predicted number of conformers was , we randomly sampled 300 points on the grid. Moreover, in the rare case that KinBot found a lower-energy conformer during the hindered rotor scans, the new lower-energy structure replaced the old one. The geometries, frequencies, and hindered rotor scans were computed at the L2 = ωB97X-D/6-311++G(d,p) level of theory. For each rotor, the energy was calculated for 24 dihedral angles separated by 15°. All degrees of freedom except the scanned dihedral were relaxed. Failed points were approximated using interpolation based on a Fourier fit. The motion along the rotors at the minimum was projected out from the Hessian to arrive at the reduced set of harmonic frequencies. Final stationary-point energies were obtained at the L3 = CCSD(T)-F12/cc-pVTZ-F12//ωB97X-D/6-311++G(d,p) level of theory.

All DFT geometry optimizations, frequency calculations, and energy calculations were performed with Gaussian 16.^21^ Input files were generated for Gaussian 16 thorugh the atomic simulation environment package^22^ as called by KinBot. PESViewer^23^ was used to visualize potential energy surfaces, which invokes RDKit^24^ for molecular structure visualization. The coupled-cluster L3 energies were obtained using Molpro.^25^ T1 diagnostics for each stationary point are provided in Table S1 of the Supporting Information.

Two conditions were defined for a well to be included. The first condition is that the barrier height to a given well is lower than 30.0 kcal/mol relative to the initial radical. The second is that the well is at least 5% of the branching fraction from another included well at the high-pressure limit at 400 or 1000 K. The two conditions selected prevent the network of reactions from becoming excessively large. Figure 4 shows the nomenclature for the radicals in the present work.

### Rate Coefficient Calculations

For each stationary point, the input parameters were taken for the lowest-energy conformer identified by KinBot. Estimates for the Lennard-Jones parameters, ε = 252 K and σ = 4.36, were calculated according to the formula for alcohols given by Jasper.^26^ The effective number of heavy atoms, *N*_eff_, was  for each of the DMO radicals. Calculations of α were conducted between 300 and 1000 K and fit to an equation of the form α_0_ × (*T*/300 K)^*n*^, which yielded α_0_ = 323 cm^–1^ and *n* = 0.53 with an RMS of 25 cm^–1^. The collision parameters for each of the radicals are identical because the connectivity is unchanged. The master equations were assembled by KinBot and solved using the MESS^27^ code. The direct solving method was used except for the R1 system, for which the low eigenvalue method was employed because of numerical instability below 400 K.

### Uncertainty Analysis

In the present work, we utilized the uncertainty analysis feature recently added to KinBot for theoretical rate coefficients. For each DMO radical PES, 100 different master equations were solved with random, independent perturbations to each of the stationary-point energies. Transition-state energies were perturbed according to a uniform distribution by a maximum of 1.0 kcal/mol. Similarly, well depths were perturbed by a maximum of 0.5 kcal/mol. The branching fractions were computed for each master equation solution at 1 atm and 20 ms from 300 to 1000 K. The major products for all 100 solutions are given in the Theoretical Yields section of the present work.

## Results and Discussion

### Potential Energy Surfaces

**Figure 5:**
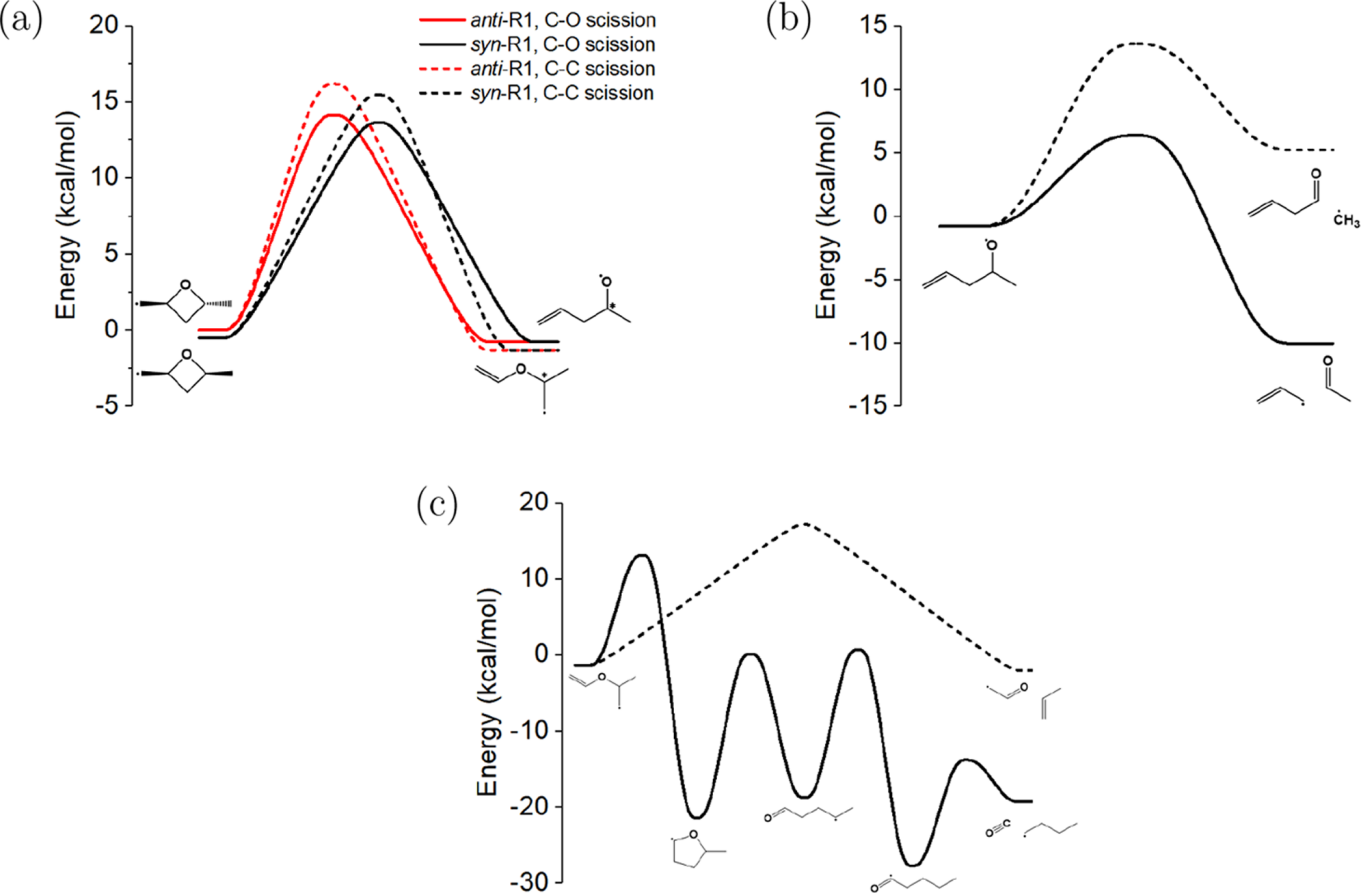
Potential energy surfaces computed at the CCSD(T)-F12/cc-pVTZ-F12 level of theory: (a) ring-opening of the primary radical, 2,4-dimethyloxetan-1-yl, R1, to either pent-1-en-4-oxy via C–O β-scission or 2-(vinyloxy)propan-2-yl via C–C β-scission; bimolecular product channels for the decomposition of (b) pent-1-en-4-oxy and (c) 2-(vinyloxy)propan-2-yl.

**Figure 6:**
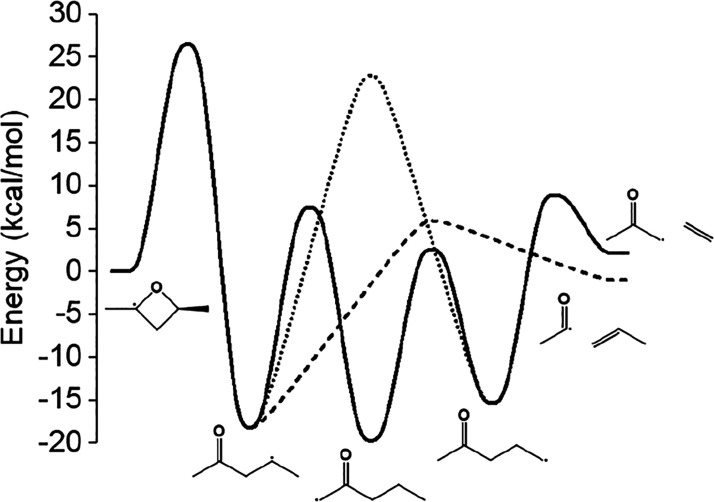
Potential energy surface computed at the CCSD(T)-F12/cc-pVTZ-F12 level of theory for the unimolecular reactions of R2. Ring-opening of the tertiary radical, 2,4-dimethyloxetan-2-yl, follows either a sequence of pentanonyl isomerization steps yielding acetonyl + ethene or acetyl + propene via C–C β-scission of penta-2-one-4-yl.

**Figure 7:**
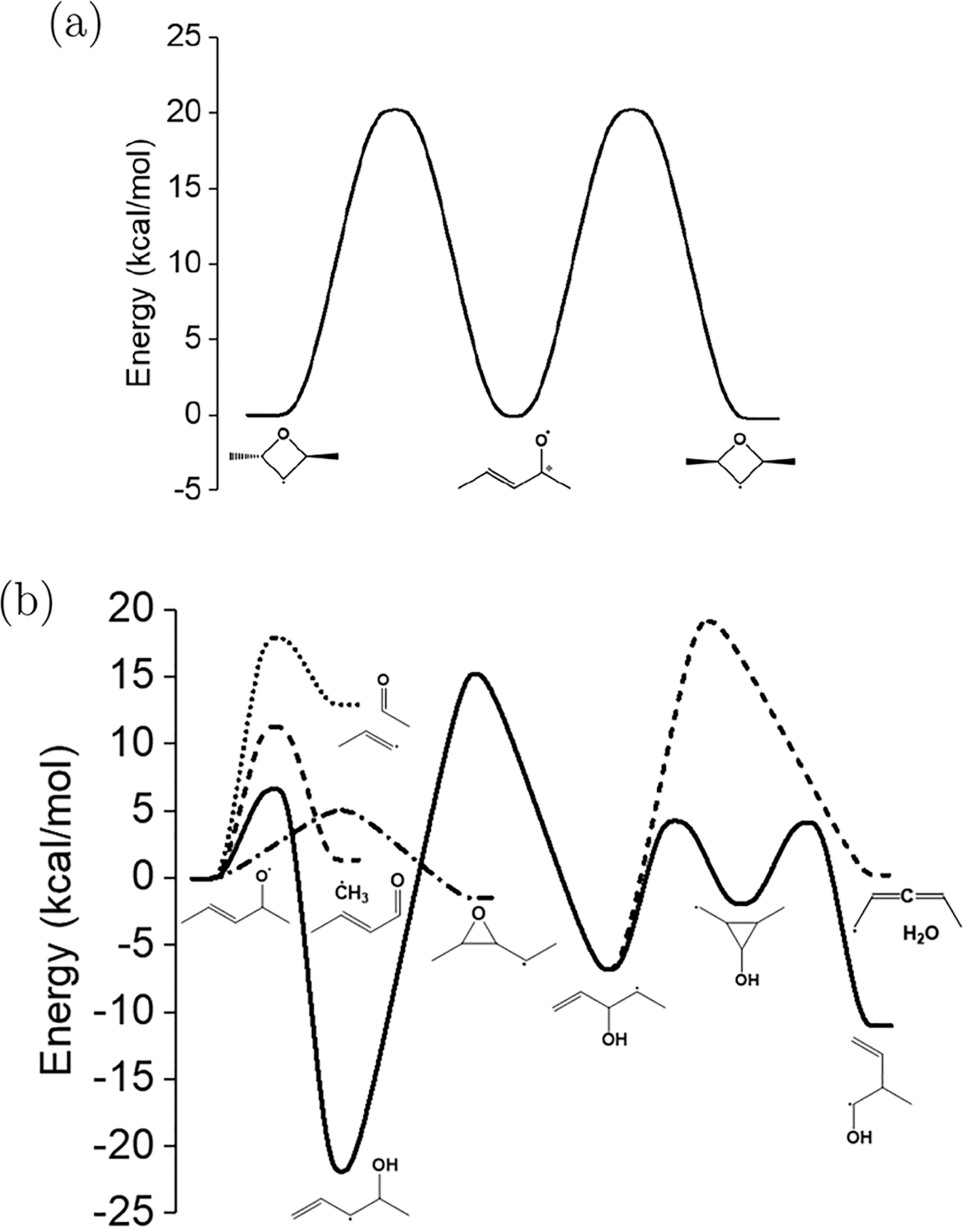
Potential energy surfaces computed at the CCSD(T)-F12/cc-pVTZ-F12 level of theory for R3. Both stereoisomers of the secondary radical, 2,4-dimethyloxetan-3-yl, undergo ring-opening to yield pent-2-en-4-oxy (a), which may then yield several bimolecular product pairs including *trans*-2-butenal + CH_3_ (b).

The potential energy surfaces for *syn*- and *anti*-R1, R2 and *syn*- and *anti*-R3 are given by Figures 5, 6, and 7, respectively. In general, T1 diagnostics of the wells were below 0.02 with the exception of the acetyl radical (0.023), while transition state T1 diagnostics were below 0.04 with the exceptions of the ring-opening step for R2 (0.042) and the β-scission of R1 ring-opened via C–C bond scission to give vinoxy and propene (0.042).

Both R1 diastereomers ring-open to produce either the unsaturated alkoxy radical, 1-pentene-4-oxy, through C–O bond scission, or the unsaturated ether radical, 2-(vinyloxy)propan-1-yl, through C–C bond scission. R2 ring-opens to produce the carbonyl alkyl radical, 2-pentanone-4-yl (Figure 6). The ring-opening pathway via C–C bond scission for R2 was excluded due to a branching ratio from the initial radical of  5%. Both R3 diastereomers ring-open only via C–O bond scission, producing the unsaturated alkoxy radical 2-pentene-4-oxy (Figure 7). Because of the symmetry of the molecule, the pathways for scission of each C–O bond are identical.

The DMO radical diastereomers for R1 and R3 are <0.5 kcal/mol different in energy. No significant difference is evident between the barrier heights for the ring-opening of the diastereomers. The barrier height for ring-opening is lowest for C–O bond scission of the R1 radicals (14 kcal/mol) and highest for C–O bond scission of the R2 radical. The barrier for ring-opening of R1 via C–C bond scission is slightly higher at 16 kcal/mol. The barrier height for ring-opening of R3 via C–O bond scission is in the middle at 20 kcal/mol. Notably, ring-opening is not significantly exothermic except for the case of R2, which forms 2-pentanone-4-yl.

On the R1 surface, the lowest-energy pathway for 2-(vinyloxy)propan-1-yl, the ring-opened radical formed via C–C bond scission, is ring closure to give 2-methyltetrahydrofuran-5-yl. The most abundant cyclic ether in the low-temperature combustion of *n*-pentane is 2-methyltetrahydrofuran.^11^ The potential energy surface shows that 2-methyltetrahydrofuran-5-yl produces CO and *n*-butyl by ring-opening, H-transfer, and decarbonylation of the α-pentanal radical intermediate. Similar decarbonylation reactions were observed in decomposition of α-aldehyde radicals^28^ and cyclic ether species,^29,30^ and further evidenced in experiments of Rajakumar et al.,^31^ which indicated an unknown source of carbon monoxide during thermal decomposition of 2-methyltetrahydrofuran in a shock tube.

Both unsaturated alkoxy radicals (1-pentene-4-oxy and 2-pentene-4-oxy from R1 and R3 ring-opening, respectively) can undergo methyl loss via β-scission, yet over relatively higher barriers compared with other pathways. The lowest-energy pathway for the ring-opened R3 radical is ring closure to form 2-methyl-3-(ethyl-1-yl)oxirane. However, 95% of the flux out of the well is the reverse reaction forming 2-pentene-4-oxy. The next-lowest-energy pathway is hydrogen transfer from C1 to the oxy radical through a six-membered transition state forming 1-penten-4-ol-3-yl. The pathway to this allylic alcohol radical is 21.9 kcal/mol exothermic. The only pathway forward for 1-penten-4-ol-3-yl is OH migration to form 1-penten-3-ol-4-yl, which has a 27.5 kcal/mol barrier.

### Rate Coefficients

**Figure 8:**
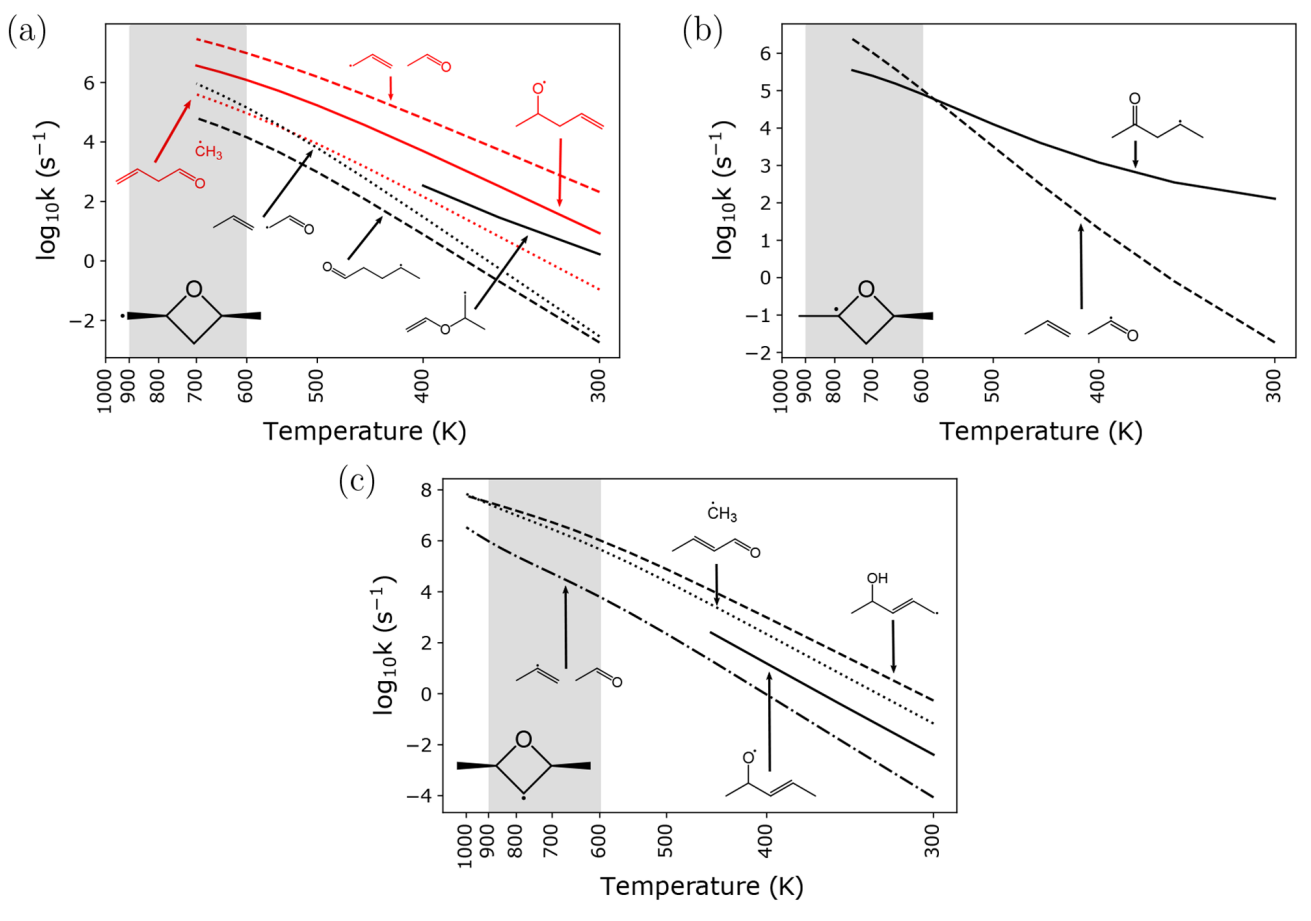
Atmospheric pressure rate coefficients for ring-opening of *syn*-R1 (a), R2 (b), and *syn*-R3 (c) are given across temperatures 300–1000 K on a logarithmic scale. Well-skipping pathways are denoted with dotted or dashed lines. In (a), red lines denote pathways that involve ring-opening by C–O bond scission while black lines correspond to pathways with ring-opening via C–C bond scission. The temperature region (600–900 K) where 2,4-dimethyloxetane has been detected experimentally by Bugler et al.^13^ is shaded in gray.

The largest rate coefficients for *syn*-R1, R2 and *syn*-R3 at atmospheric pressure are given by Figure 8. The rate coefficients for *anti*-R1 and *anti*-R3 are nearly identical and are given in Figures S2 and S5 of the Supporting Information, respectively. For R1, the low-eigenvalue method was used instead of directly solving the master equation because of numerical instability at low temperatures for *anti*-R1.

**Figure 9:**
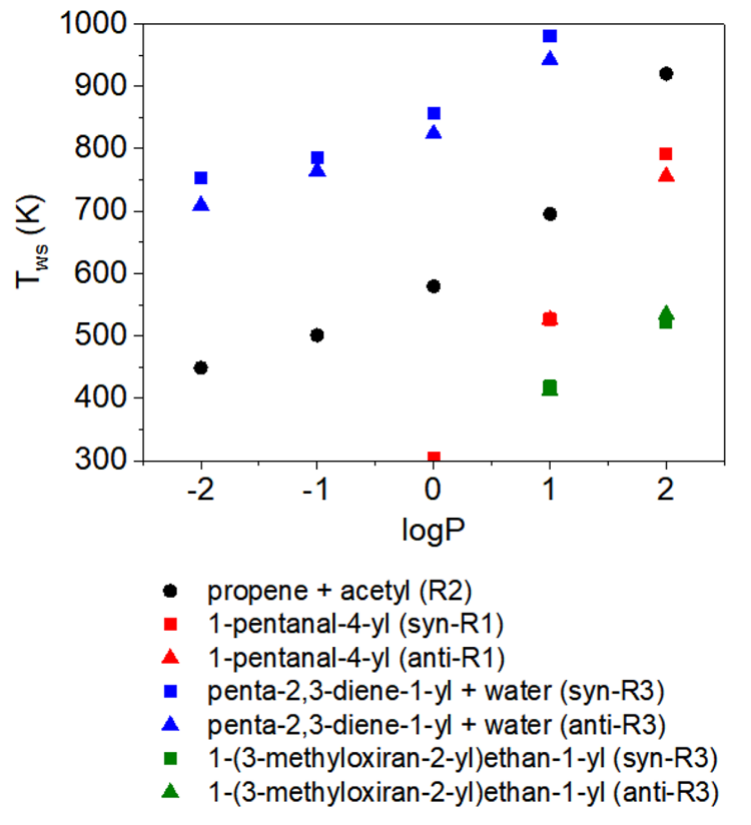
Pressure dependence of the temperature at which the rate coefficient of well-skipping for each pathway is equal to the rate coefficient of the stepwise reaction: the well-skipping crossover temperature, *T*_ws_. Above *T*_ws_, well skipping is faster than the stepwise reaction. Below *T*_ws_, the stepwise reaction is faster than well skipping. Reactions not included typically favored well skipping across the entire temperature and pressure range.

The present work predicts the rate coefficient for methyl loss from 1-pentene-4-oxy at the high-pressure limit and 1000 K to be 1.44 × 10^11^ s^–1^, which is more than 4 times slower than the 10^12^ s^–1^ rate coefficient assigned across the entire temperature range in the *n*-pentane mechanism from Bugler et al.^11^ At 600 K, the high-pressure-limit rate coefficient for this reaction is more than 500 times slower than the prescribed rate coefficient. For the R1 diastereomers, the rate coefficient for the well-skipping reaction to ring-open via C–O bond scission and β-scission is approximately an order of magnitude larger than the stepwise ring-opening by C–O bond scission across the entire temperature range. The rate coefficiet for stepwise ring-opening via C–C bond scission is larger than the than the corresponding well-skipping reactions at low temperature (400 K), yet the stepwise rate coefficient disappears with increasing temperature.

For the R3 diastereomers, the well-skipping reaction to the allylic radical 1-penten-4-ol-3-yl via C–O ring-opening and H-transfer is favored across the entire temperature range at atmospheric pressure, by approximately 2 orders of magnitude than the stepwise ring-opening reaction that gives 2-penten-4-oxy. The rate coefficient for the reaction that produces 2-butenal + methyl by skipping the 2-penten-4-oxy and 1-penten-4-ol-3-yl wells overtakes the rate coefficient for the reaction producing 1-penten-4-ol-3-yl between 900 and 1000 K. The rate coefficient for the reaction producing propene-2-yl + acetaldehyde, one of the products proposed by Bugler et al.,^11^ is approximately 2–4 orders of magnitude smaller than the largest rate coefficient across the entire temperature range.

The concept of the well-skipping crossover temperature (*T*_ws_) is clearly demonstrated in (b) of Figure 8, where the rate coefficient for ring-opening R2 to 2-pentanone-4-yl intersects the rate coefficient for ring-opening R2 and β-scission of 2-pentanone-4-yl in one step at approximately 600 K. Above 600 K, the well-skipping reaction is favored over the stepwise reaction, and below 600 K, the stepwise reaction is favored. As expected, the well-skipping crossover temperature always increases with pressure. Diastereomer pathways in this system have approximately the same well-skipping crossover temperatures across the entire pressure range. The largest difference between well-skipping crossover temperatures for diastereomer pathways are for the R3 pathway to penta-2,3-diene-1-yl + water. The well-skipping crossover temperatures are approximately 20–50 K higher for the *syn* diastereomer compared to those for the *anti* diastereomer from 0.01 to 10 atm.

### Theoretical Yields

**Figure 10:**
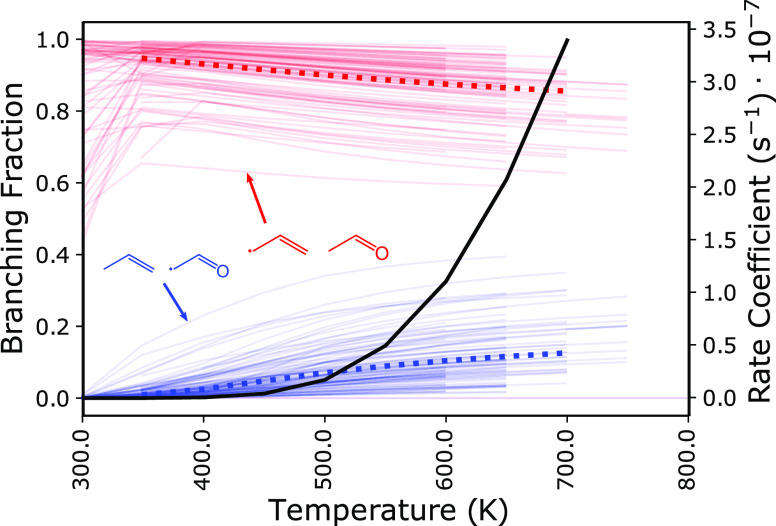
Branching fractions for *syn*-R1 at 1 atm and 20 ms are given between 300 and 800 K. Uncertainties are represented by including results from 100 random perturbations in each stationary-point energy. Some lines are truncated due to disappearing rate coefficient values at high temperatures where the rate coefficients are larger than collisional relaxation. The major product is acetaldehyde + allyl (red), which originates from β-scission of the (C–O) ring-opened radicals and has a branching fraction of between 0.6 and 1.0 across all temperatures shown. Propene and the vinoxy radical from the other (C–C) ring-opened radical are minor products (blue) and have a branching fraction of between 0.0 and 0.35, which increases with temperature. The nominal values are given by the bold, dotted lines. The results for *anti*-R1 are nearly identical and are included in the Supporting Information.

The branching fractions for *syn*-R1 are given in Figure 10. The products with the highest branching fractions across the entire temperature range are allyl + acetaldehyde from ring-opening by C–O bond scission followed by β-scission, which is the lowest-energy product channel. The products with the second-highest branching fraction are vinoxy + propene from ring-opening by C–C bond scission followed by β-scission. While the ring-closure pathway forming 2-methyltetrahydrofuran-5-yl is the lowest-energy pathway for 2-(vinyloxy)propan-1-yl, β-scission to form vinoxy + propene is entropically favored. Although the branching fractions to the bimolecular product channel, CO + 1-butyl, are negligible (5%) at 1 atm, flux to the 2-methyltetrahydrofuranyl may facilitate O_2_ addition and open the potential for products from such a crossover reaction as postulated in Doner et al.^14^ The uncertainty for the branching fractions of R1 are rather high because of the larger number of stationary-point energies involved compared to, for example, R2.

**Figure 11:**
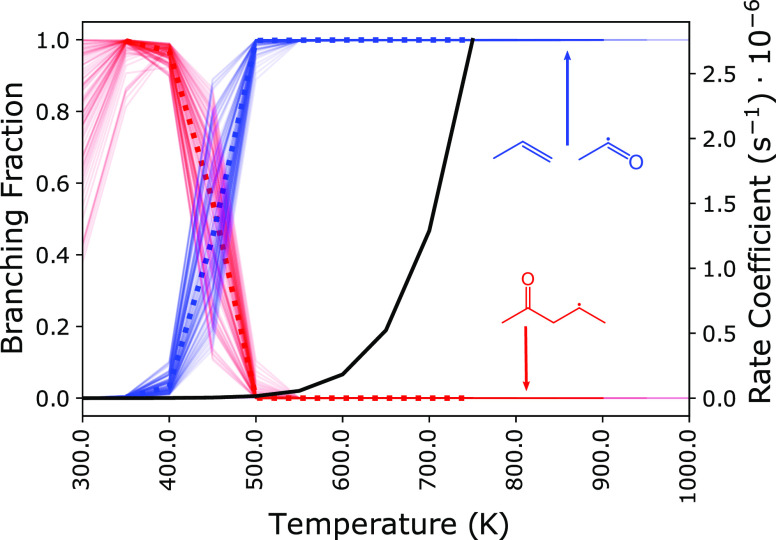
Branching fractions for R2 at 1 atm and 20 ms are given between 300 and 1000 K. Uncertainties in the yields are represented by including results from 100 random perturbations to each stationary-point energy. Some lines are truncated due to disappearing values when well-skipping becomes dominant at high temperatures. Above 500 K, the branching is approximately 100% propene + acetyl from the lowest-energy pathway. Below 500 K, the overall consumption rate (black) for R2 is low, yet the yield is mostly 2-petnanone-4-yl, the precursor to propene + acetyl. The nominal values are given by the bold, dotted lines.

The branching fractions for R2 at 1 atm from 300 to 1000 K are given by Figure 11. Above 500 K, the R2 radical produces approximately 100% propene + acetyl radical formed through a 5.9 kcal/mol barrier from the (C–O) ring-opened radical, pentanone-4-yl. Below 500 K, the branching fraction is largest for 2-pentanone-4-yl. However, the consumption rate of R2 below 500 K is negligible ( 10^4^ s^–1^).

**Figure 12:**
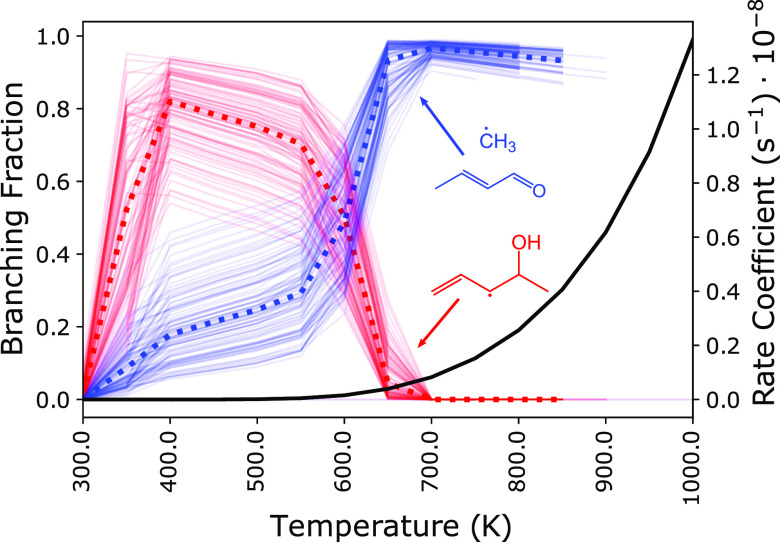
Branching fractions for *syn*-R3 at 1 atm and 20 ms are given between 300 and 900 K. Uncertainties are represented by including results from 100 random perturbations to each stationary-point energy. Some lines are truncated due to disappearing values when well-skipping becomes dominant at high temperatures. At low temperatures (*T* 600 K), the majority of the yield (50–90%) is 1-penten-3-yl-4-ol radical (red), which exists in a deep energetic well due to the allylic character. However, the consumption of *syn*-R3 (black) below 600 K is negligible. At higher temperatures (*T* 600 K), the majority of the yield (90–100%) is *trans*-2-butenal + methyl (blue), which originates from β-scission of the (C–O) ring-opened radical. The nominal values are given by the bold, dotted lines. The branching fractions for *anti*-R3 are nearly identical and included in the Supporting Information.

At temperatures above 600 K, the R3 radicals produce mostly 2-butenal + CH_3_, which is the second-lowest-energy pathway (Figure 12). The aldehyde 2-butenal is present in the current mechanism for *n*-pentane combustion, and the mole fraction profile at 10 atm shows that it is underpredicted in Bugler et al.^11^ from approximately 850 to 1000 K. However, at 1 atm, no speciation data is available. Furthermore, the oxidation of 2-butenal was recently examined by Liu et al.^32^ in JSR experiments at atmospheric pressure between 500 and 850 K. Below 600 K, 2-pentene-4-oxy mostly undergoes H-transfer to form 1-penten-2-yl-4-ol, which sits in a deep energetic well. Therefore, 2-butenal is more likely to react with O_2_, especially at high pressures.

## Conclusions

In the present work, the mechanisms of the unimolecular decomposition of five radicals obtained from the stereoisomers of 2,4-dimethyloxetane were explored using KinBot. The calculated rate coefficients add crucial information to support the chemical kinetics modeling by providing constraints on the balance between unimolecular decomposition and bimolecular reactions of alkyl-substituted cyclic ethers, which are direct products of QOOH. The products following the H-abstraction of 2,4-dimethyloxetane proposed by Bugler et al.^13^ (acetyl + propene and allyl + acetaldehyde) were significant pathways in the present work, making up 100% of the yield of the tertiary 2,4-dimethyloxetan-2-yl radical and 85–90% of the yield of the R1 diastereomers under the selected conditions. However, five additional products were found. One of these, crotonaldehyde + CH_3_, makes up  90% of the yield for the 2,4-dimethyloxetan-3-yl diastereomers at atmospheric pressure over 650 K.

Overall, the consumption reactions of 2,4-dimethyloxetanyl radicals are more complex than prescribed in chemical kinetics mechanisms, particularly since ring-opening rate coefficients compete with O_2_ addition. The tertiary radical 2,4-dimethyloxetan-2-yl encounters the largest barrier height for ring-opening, which may increase the potential for bimolecular reactions, such as with O_2_. The calculations indicate that tertiary peroxy radicals are likely favored because ring-opening rate coefficients for R2 (2,4-dimethyloxetan-2-yl) are approximately 1 order of magnitude lower than for primary and secondary radicals, which are both on the order of 10^7^ s^–1^ at combustion-relevant temperatures.

Although stereochemistry shows a dramatic effect on the kinetics of peroxy radicals derived from O_2_ addition to 2,4-dimethyloxetanyl,^14^*syn* and *anti* isomers undergo unimolecular decomposition at similar rates because the methyl groups act as spectators during ring-opening, after which the stereochemistry of the starting radical is lost. However, the kinetics of 2,4-dimethyloxetanyl isomers are complicated by the pressure-dependent competition between well skipping and stepwise reactions. As such, special care is required when incorporating such reactions into a larger combustion mechanism.

To complete the description of the fate of 2,4-dimethyloxetane in combustion systems under low-temperature conditions, theoretical rate coefficients for H-abstraction from each carbon by radical species such as OH, HO_2_, CH_3_, and CH_3_O are still required, as well as the unimolecular decomposition rate coefficient of the closed-shell stereoisomers. Direct speciation experiments on stereoisomers of 2,4-dimethyloxetane are also imperative to confirm the reaction mechanisms.

## Supporting Information Available

Rate coefficients, branching fractions, and T1 diagnostics (PDF)
MESS input files (ZIP)
