# Supplementary material for: Unimolecular Reactions of 2,4-Dimethyloxetanyl Radicals
Source: J Phys Chem A. 2023 Mar 10;127(11):2591–600. doi: 10.1021/acs.jpca.2c08290 (PMC10041641; doi:10.1021/acs.jpca.2c08290)
Supplement: Supplementary file 1 — jp2c08290_si_001.pdf [file jp2c08290_si_001.pdf]

Unimolecular Reactions of 2,4-Dimethyloxetanyl Radicals

Anna C. Doner,† Judit Zádor,‡ and Brandon Rotavera∗,†,¶

†Department of Chemistry, University of Georgia, Athens, GA, USA
‡Combustion Research Facility, Sandia National Laboratories, Livermore, CA, USA
¶College of Engineering, University of Georgia, Athens, GA, USA

Rate Coefficients

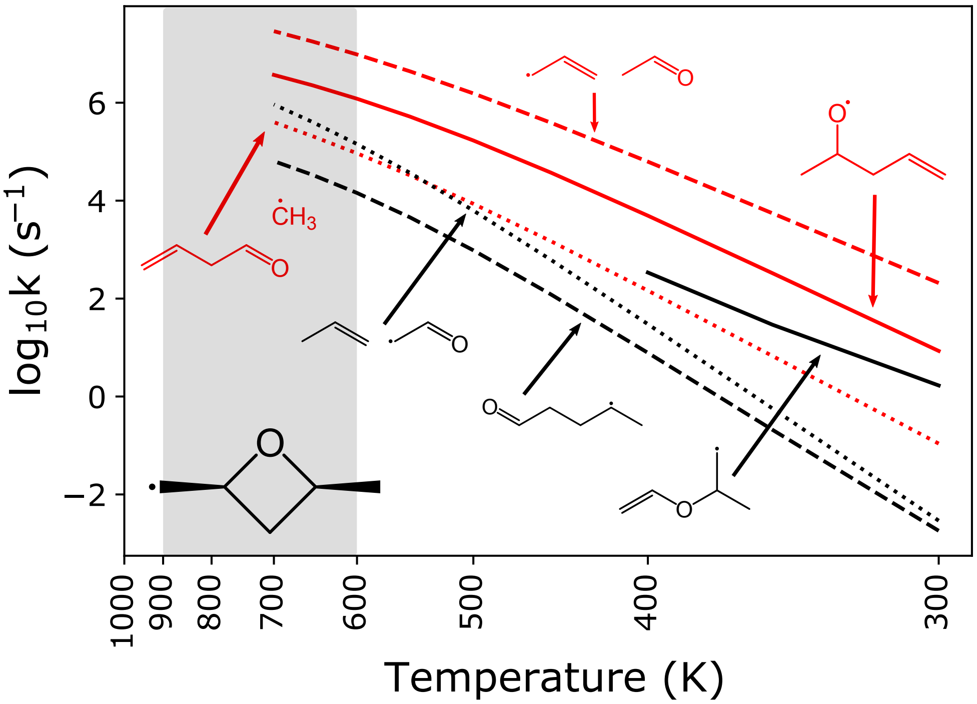

Figure S1 *syn*-2,4-dimethyloxetan-1-yl

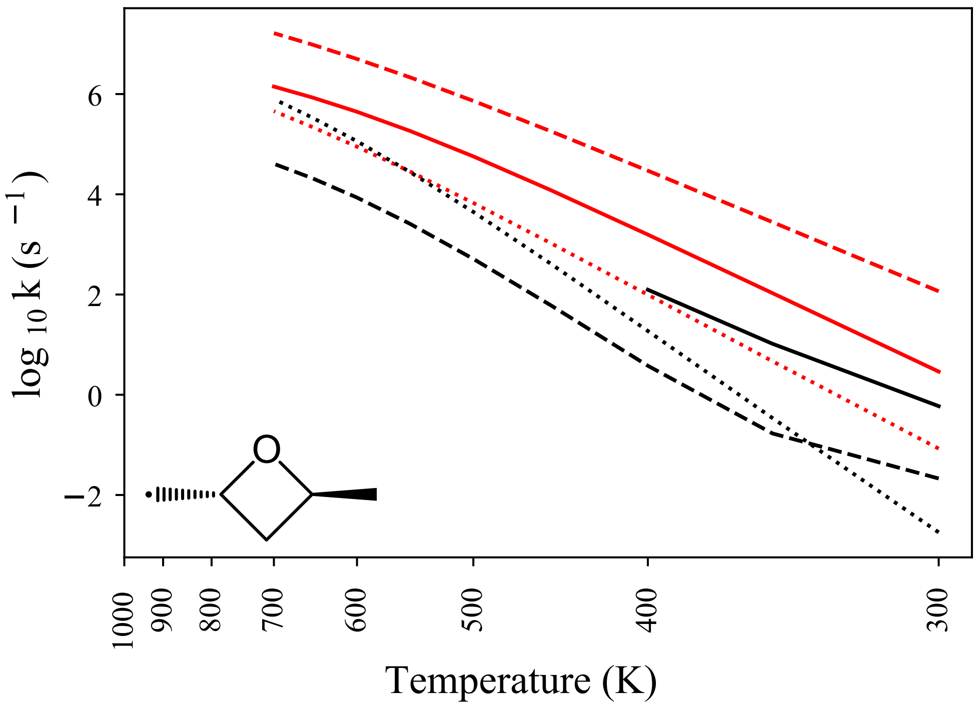

Figure S2 *anti*-2,4-dimethyloxetan-1-yl

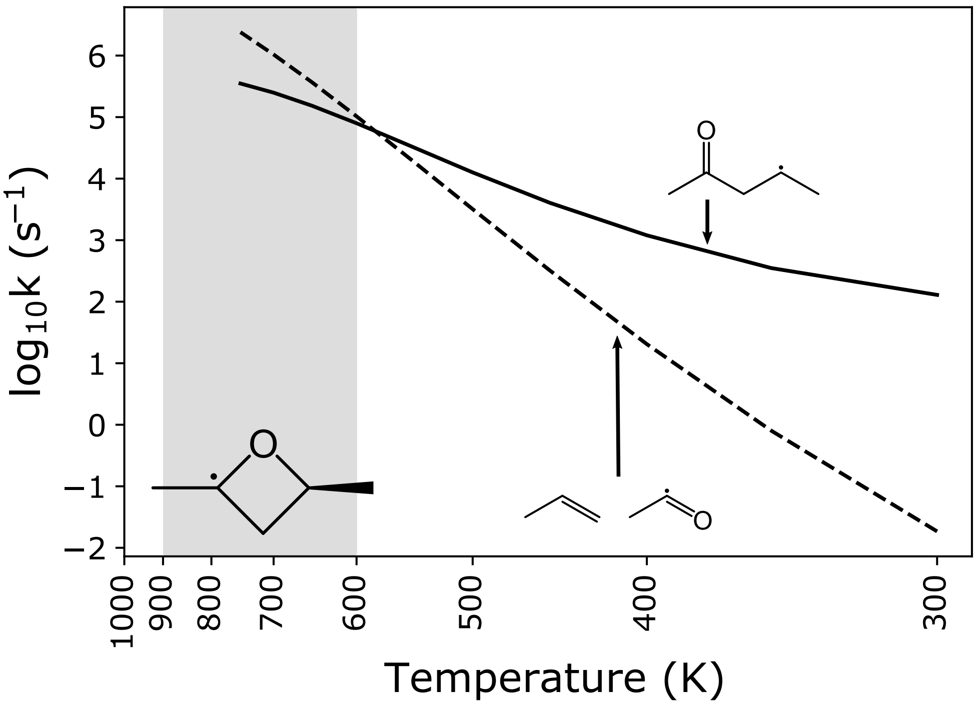

Figure S3 2,4-dimethyloxetan-2-yl

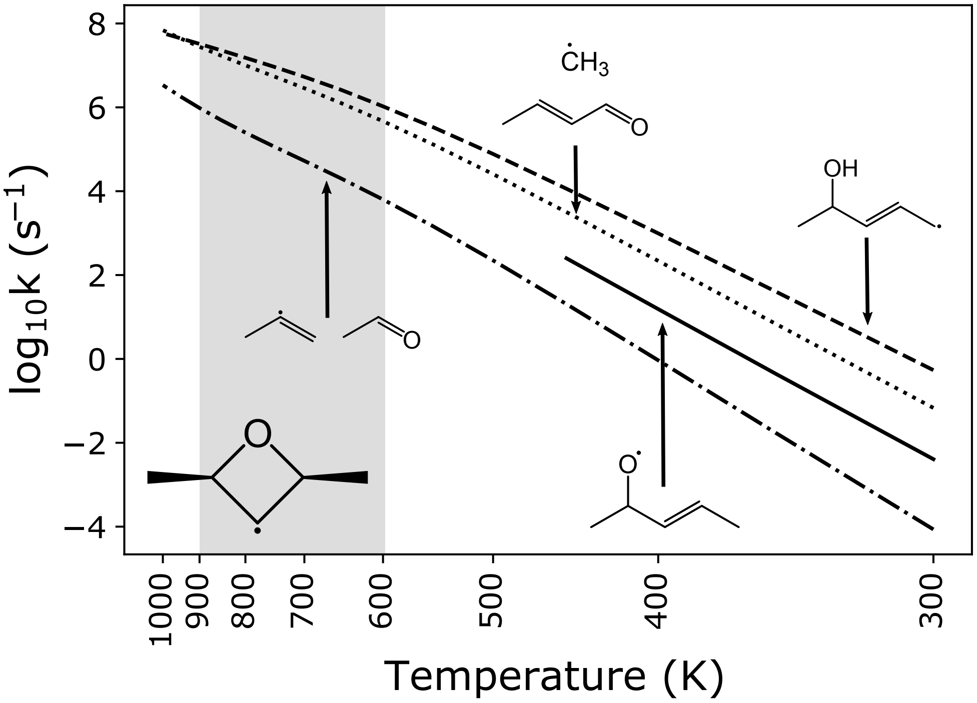

Figure S4 *syn*-2,4-dimethyloxetan-3-yl

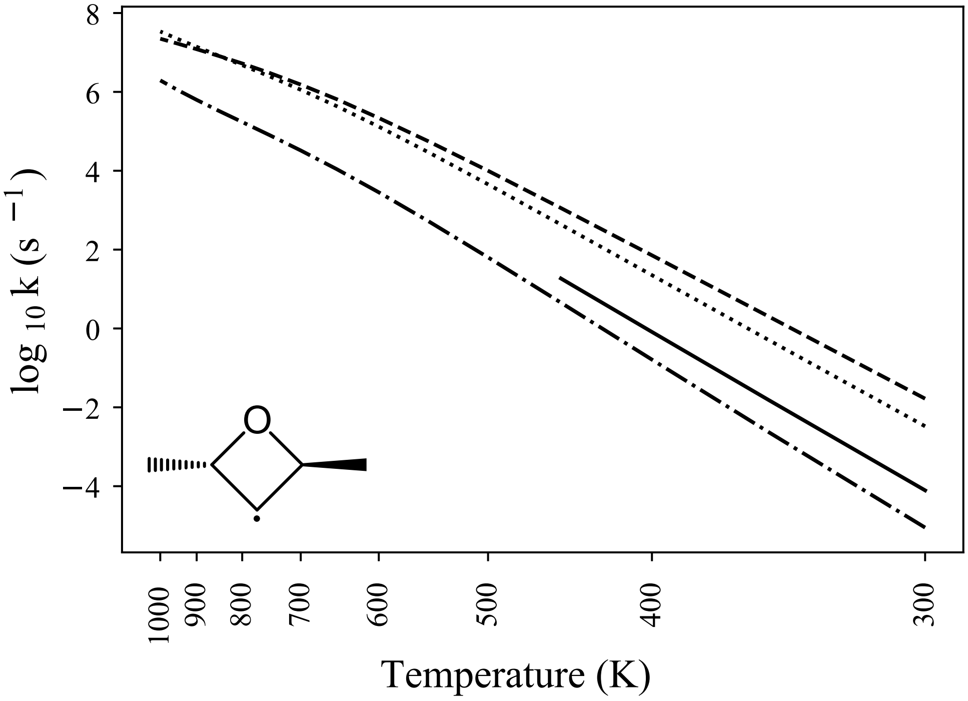

Figure S5 *anti*-2,4-dimethyloxetan-3-yl

Branching Fractions

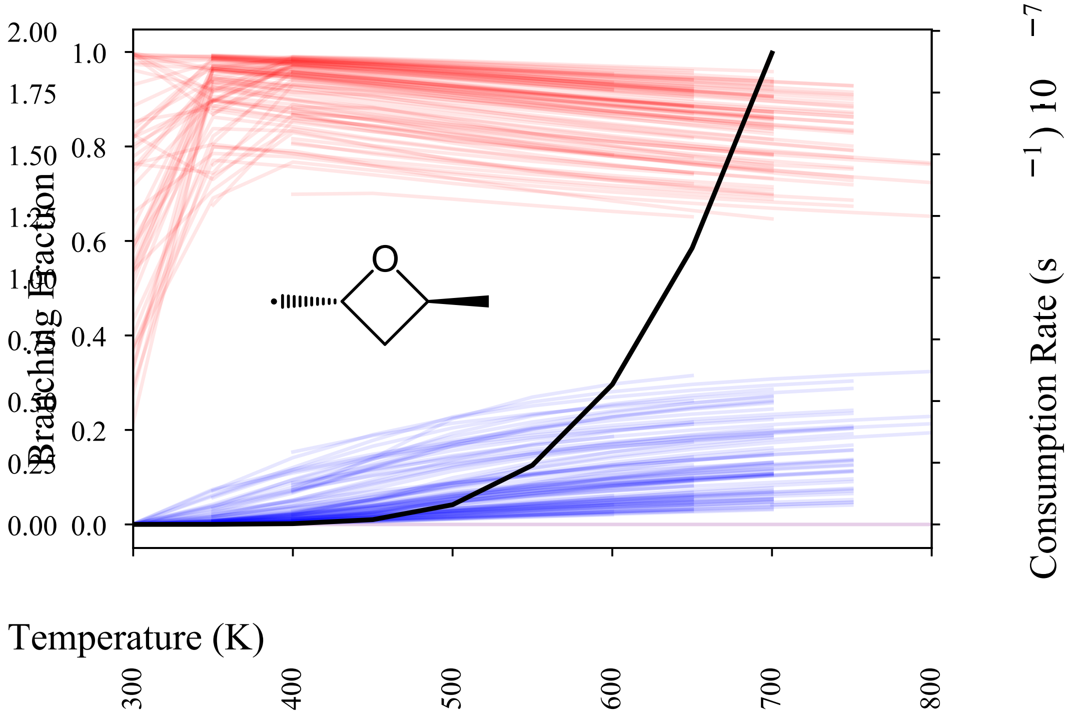

Figure S6 *anti*-2,4-dimethyloxetan-1-yl

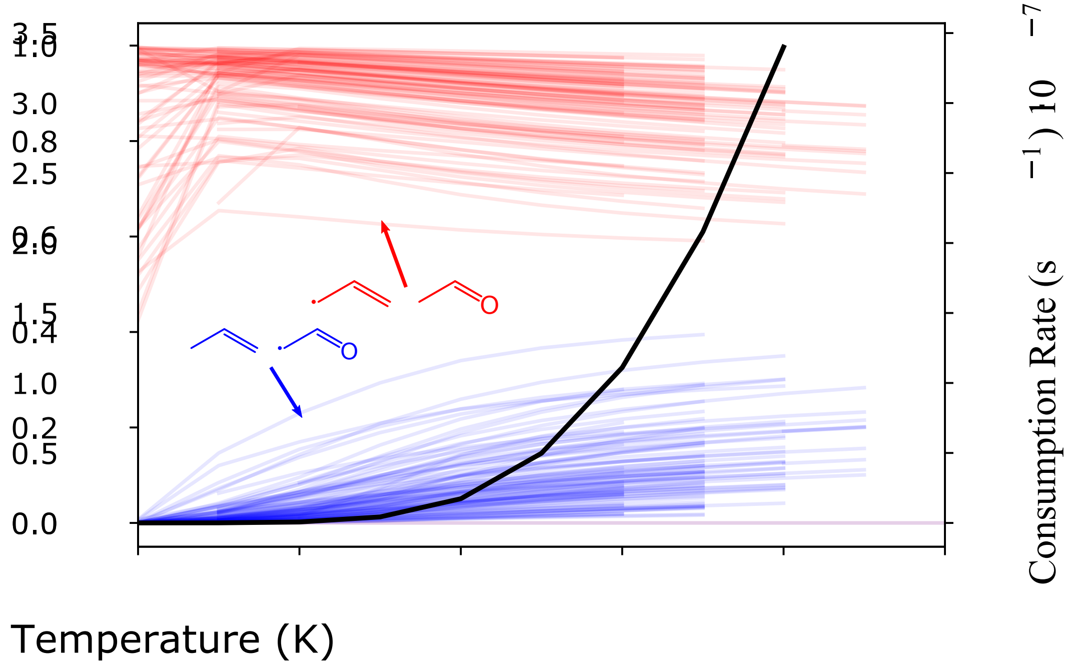

Figure S7 *syn*-2,4-dimethyloxetan-1-yl

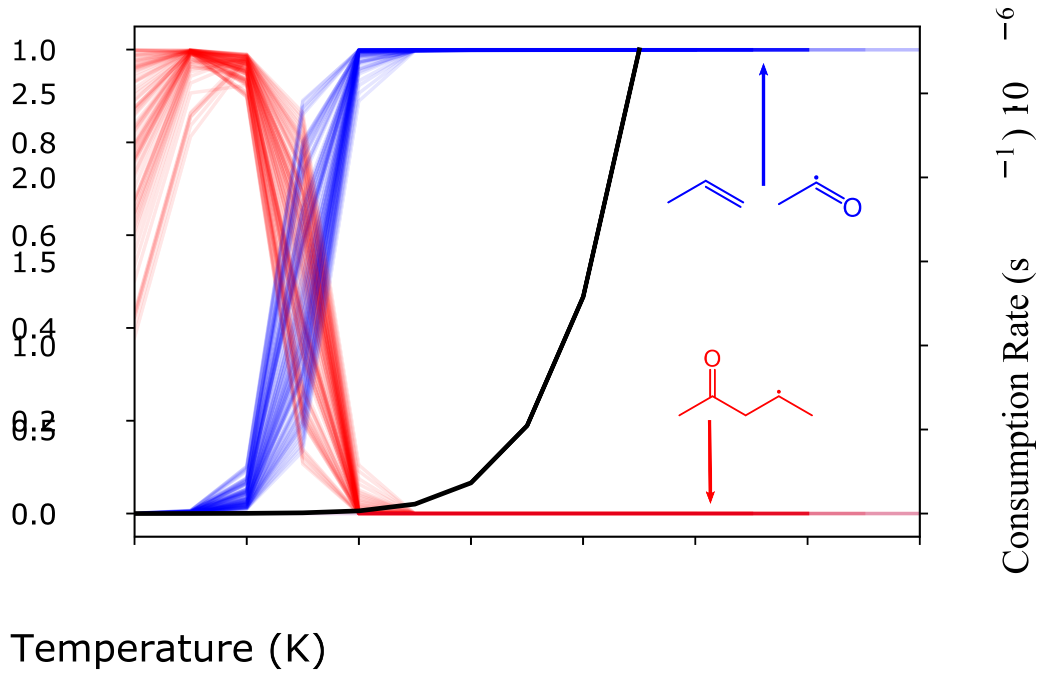

Figure S8 2,4-dimethyloxetan-2-yl

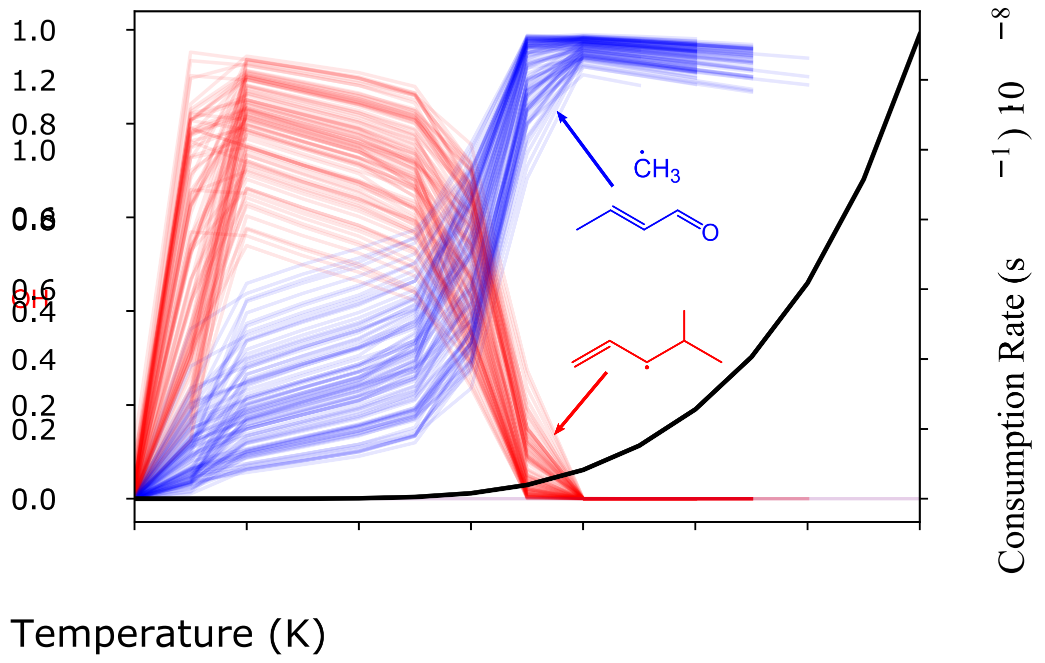

Figure S9 *syn*-2,4-dimethyloxetan-3-yl

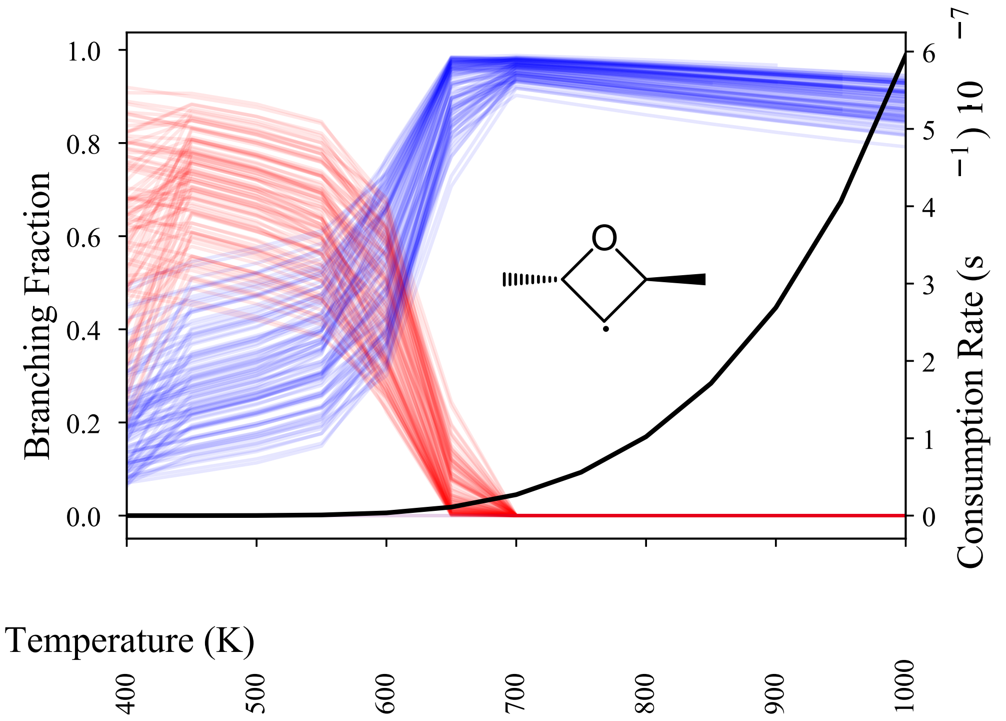

Figure S10 *anti*-2,4-dimethyloxetan-3-yl

T1 Diagnostics

Table S1 T1 Diagnostics

CHEMID	T1 diagnostic
`10000000000000000002	0.000000000
`15039006000000000002	0.006346200
`28028000000000000001	0.017651410
`411131060600080000002	0.011019320
`42078056034000000001	0.016174230
`421261230750120000001	0.010778950
`43091086038000000002	0.023005310
`431391510850120000002	0.010423740
`44104103057000000001	0.013920240
`571892291690850120002	0.010128410
`701782141760940380001	0.013423680
`842282792471801070511	0.013058750
`842282812431721110571	0.012841500
`842282922601800940381	0.012839500
`842523924764763560921	0.012452480
`842563784504423141381	0.012144420
`852413072811971130512_intra_H_migration_1_4	0.021141670
`852413072811971130512_intra_H_migration_1_7	0.019646940
`852413072811971130512_Intra_R_Add_ExoTetCyclic_F_1_6_7	0.020772780
`852413072811971130512_intra_R_migration_1_2	0.024536090
`852413072811971130512	0.016353260
`852413072811971130512_r12_insertion_R_11_10_2	0.016510970
`852413072811971130512_r12_insertion_R_2_10_11	0.021044200
`852413072811971130512_r12_insertion_R_2_10_1	0.016269280
`852413072811971130512_R_Addition_MultipleBond_1_10_11	0.020045180
`852413092621971240572	0.017705820
`852413092641891300572	0.013204660
`852413092751971110572_intra_H_migration_1_4	0.017084220
`852413092751971110572_intra_H_migration_3_11	0.016780330
`852413092751971110572_intra_H_migration_3_14	0.019727170
`852413092751971110572_intra_H_migration_3_4	0.014229810
`852413092751971110572_intra_H_migration_3_7	0.012262160
`852413092751971110572	0.013241840
`852413092751971110572_r12_insertion_R_13_1_14	0.052780400
`852413092751971110572_R_Addition_MultipleBond_1_10_2	0.015364350
`852413092751971110572_R_Addition_MultipleBond_3_2_10	0.020011820
`852413092751971110572_R_Addition_MultipleBond_3_6_7	0.014819590
`852413093201971110122	0.012925870
`852413132601871260632	0.017411690
`852413202811971130382	0.012745790

`852413243221821090122_intra_H_migration_10_15	0.034147420
`852413243221821090122_intra_H_migration_1_15	0.016442530
`852413243221821090122_intra_H_migration_13_11	0.016454380
`852413243221821090122_intra_H_migration_13_4	0.024346650
`852413243221821090122_intra_H_migration_13_7	0.017228900
`852413243221821090122_intra_R_migration_13_1	0.035221120
`852413243221821090122_intra_R_migration_13_6	0.018725110
`852413243221821090122	0.017352260
`852413243221821090122_r12_insertion_R_13_3_2	0.018085900
`852413243221821090122_r12_insertion_R_14_1_10	0.024768480
`852413243221821090122_r12_insertion_R_3_6_7	0.018725120
`852452933111831340242	0.016219660
`852452942581891260722	0.014018830
`852453052751971110532	0.014076810
`852453062961921310202_intra_H_migration_2_11	0.013162640
`852453062961921310202_intra_H_migration_2_14	0.013378930
`852453062961921310202_intra_R_migration_2_13	0.041722920
`852453062961921310202	0.012507930
`852453083091981180122	0.013522490
`852453113291731160162	0.013246930
`852453203181861090122	0.011951520
`852453223351880920082	0.013045600
`852694064864873611502_h2_elim_12_14	0.016326370
`852694064864873611502_intra_H_migration_1_7	0.014266820
`852694064864873611502_Korcek_step2_10_4	0.016312430
`852694064864873611502	0.014831720
`852694064864873611502_r12_insertion_R_3_2_10	0.023310740
`852694064864873611502_R_Addition_MultipleBond_1_10_11	0.014444960
`852694104784873691462	0.011776530
`852694214845023821222	0.011551100
`852694215404703600442	0.012064860
`852694215404703600442_r13_insertion_ROR_1_2_3_13	0.020211990
`20020000000000000001	0.006088250
`280760560080000000001	0.011496730
`430910730510000000002	0.021241990
`571411531500120000002	0.019277130
`832393644334022060572	0.018033950
`842282702691721350181	0.013760420
`842282812991671050121	0.012984990
`852412982902021410182	0.016885590
`852412983141841350182_intra_H_migration_11_7	0.019453930
`852412983141841350182_intra_H_migration_1_4	0.033867780
`852412983141841350182_intra_H_migration_3_4	0.016051830
`852412983141841350182	0.013312550

`852412983141841350182_r12_insertion_R_4_2_3	0.016340380
`852412983141841350182_R_Addition_MultipleBond_1_10_13	0.024571070
`852412983141841350182_R_Addition_MultipleBond_1_2_4	0.016338990
`852412983141841350182_R_Addition_MultipleBond_3_2_1	0.021113650
`852412983141841350182_R_Addition_MultipleBond_3_6_7	0.014898040
`852413093071971240122_intra_H_migration_10_4	0.013429160
`852413093071971240122_intra_H_migration_1_12	0.014416510
`852413093071971240122_Intra_RH_Add_Exocyclic_F_10_12	0.014416620
`852413093071971240122_Intra_RH_Add_Exocyclic_F_10_7	0.014595870
`852413093071971240122_Intra_RH_Add_Exocyclic_F_11_12	0.023542210
`852413093071971240122_Intra_RH_Add_Exocyclic_F_11_7	0.019363380
`852413093071971240122	0.016429970
`852413093071971240122_r12_insertion_R_1_2_4	0.017499950
`852413093071971240122_r12_insertion_R_2_1_11	0.018098920
`852413093201971110122_h2_elim_7_8	0.015901600
`852413093201971110122_intra_H_migration_11_7	0.047884770
`852413093201971110122_intra_H_migration_6_7	0.012543550
`852413093201971110122_r12_insertion_R_3_2_4	0.020773760
`852413093201971110122_r12_insertion_R_4_2_1	0.015969810
`852413093201971110122_R_Addition_MultipleBond_6_3_2	0.020773580
`852452942862061410182	0.022146250
`852452943101881350182	0.012454820
`852452943251901200162	0.014216740
`852453053162011110122	0.011983470
`852693955364404040602	0.015332360
`852693955364404040602_r12_insertion_R_2_3_11	0.042315450
`852693955364404040602_r13_insertion_ROR_1_2_3_11	0.042321400
`170170000000000000002	0.007760790
`180340020000000000001	0.008702890
`270630410040000000002	0.017281780
`411131080600060000002	0.015123170
`581581661200720060001	0.011812880
`671871971491170750122	0.022019310
`671871971581120710122	0.014376910
`682002231791270750121	0.011389160
`701782031571050570001	0.013971010
`701821991571050530041	0.012701480
`842282923042180500001	0.012873490
`842322662652320850121	0.011939560
`842322662792400710041	0.012025500
`842322662801781200161	0.012326860
`852413132901871410182_intra_H_migration_9_3	0.022049150
`852413132901871410182_Intra_R_Add_ExoTetCyclic_F_9_2_1	0.014789740
`852413132901871410182_Intra_R_Add_ExoTetCyclic_F_9_6_8	0.013087360

`852413132901871410182	0.018406300
`852413132901871410182_r12_insertion_R_11_7_15	0.019634810
`852413132901871410182_r12_insertion_R_11_7_6	0.019444770
`852453053192460710042	0.014184910
`852453053192460710042_r12_insertion_R_15_7_2	0.016183430
`852453053192460710042_r12_insertion_R_2_7_15	0.019637260
`852453053192460710042_R_Addition_MultipleBond_6_7_11	0.020500920
`852453053192460710042_R_Addition_MultipleBond_6_9_10	0.021047860
`852453092972420850122_Intra_R_Add_ExoTetCyclic_F_7_2_3	0.019211560
`852453092972420850122	0.012179990
`852453092972420850122_r12_insertion_R_2_6_8	0.019592920
`852453092972420850122_r12_insertion_R_7_6_9	0.027511120
`852453092972420850122_r12_insertion_R_9_6_8	0.025759520
`852453092972420850122_R_Addition_MultipleBond_2_6_8	0.016105520
`852453092972420850122_R_Addition_MultipleBond_7_6_9	0.027483940
`852453093031931240162_h2_elim_3_15	0.016383760
`852453093031931240162_h2_elim_8_10	0.025759450
`852453093031931240162_intra_H_migration_1_10	0.021561370
`852453093031931240162	0.016628600
`852453093031931240162_r12_insertion_R_7_11_12	0.020090450
`852453093031931240162_r12_insertion_R_9_7_15	0.025619280
`852694104544172771082_intra_H_migration_9_3	0.011906390
`852694104544172771082	0.013484210
`852694104544172771082_r12_insertion_R_6_7_9	0.013087360
`852694105084623900482	0.012436060
`852694105084623900482_r13_insertion_ROR_7_6_2_9	0.014568380
`852694315705282220222	0.013268420
`852694315705282220222_r12_insertion_R_2_6_9	0.018695760
`852694315705282220222_R_Addition_MultipleBond_1_2_7	0.019209380

for MESS input and output files, see zipped folder “MESS Files.zip”
